# Genome-Wide Association Mapping of Yield and Grain Quality Traits in Winter Wheat Genotypes

**DOI:** 10.1371/journal.pone.0141339

**Published:** 2015-10-23

**Authors:** W. Tadesse, F. C. Ogbonnaya, A. Jighly, M. Sanchez-Garcia, Q. Sohail, S. Rajaram, M. Baum

**Affiliations:** International Center for Agricultural Research in the Dry Areas (ICARDA), Beirut, Lebanon; Pennsylvania State University, UNITED STATES

## Abstract

The main goal of this study was to investigate the genetic basis of yield and grain quality traits in winter wheat genotypes using association mapping approach, and identify linked molecular markers for marker assisted selection. A total of 120 elite facultative/winter wheat genotypes were evaluated for yield, quality and other agronomic traits under rain-fed and irrigated conditions for two years (2011–2012) at the Tel Hadya station of ICARDA, Syria. The same genotypes were genotyped using 3,051 Diversity Array Technologies (DArT) markers, of which 1,586 were of known chromosome positions. The grain yield performance of the genotypes was highly significant both in rain-fed and irrigated sites. Average yield of the genotypes ranged from 2295 to 4038 kg/ha and 4268 to 7102 kg/ha under rain-fed and irrigated conditions, respectively. Protein content and alveograph strength (W) ranged from 13.6–16.1% and 217.6–375 Jx10-4, respectively. DArT markers wPt731910 (3B), wPt4680 (4A), wPt3509 (5A), wPt8183 (6B), and wPt0298 (2D) were significantly associated with yield under rain-fed conditions. Under irrigated condition, tPt4125 on chromosome 2B was significantly associated with yield explaining about 13% of the variation. Markers wPt2607 and wPt1482 on 5B were highly associated with protein content and alveograph strength explaining 16 and 14% of the variations, respectively. The elite genotypes have been distributed to many countries using ICARDA’s International system for potential direct release and/or use as parents after local adaptation trials by the NARSs of respective countries. The QTLs identified in this study are recommended to be used for marker assisted selection after through validation using bi-parental populations.

## Introduction

Wheat is the most important and strategic food crop at global level in general and in the Central and West Asia and North Africa (CWANA) region in particular as the region have the highest average demand of wheat (191 kg/capita/year). The CWANA region grows wheat on 55 million hectares and produces about 112 million tons on annual basis [1]. The average wheat productivity in the region (2.5 t/ha) is very low as compared to the global average (3 t/ha) mainly due to yellow rust, drought, and heat stresses associated with climate change. The effect of climate change is also evident on the quality of wheat as increased heat results in shriveled wheat grains [2].

In most developing countries, apart from grain yield and disease resistance, grain quality was not a strong criterion of variety selection. However, things have changed as a result of changing food habits, increasing urbanization and trends towards raising middle class society. As a consequence, some developing National Agricultural Research Systems (NARS) are critically looking for better quality varieties suiting for preparation of different end products. Identification and utilization of molecular markers for marker assisted selection would enhance the development of widely adapted and high yielding varieties with resistance/tolerance to abiotic and biotic resistance and acceptable level of end use quality [3–5].

Association mapping (AM) using phenotypic and genotypic data of association panels has become an important approach in identifying molecular markers (QTLs) linked to traits of interest for potential use in marker assisted selection for the fact that it enables to use diverse set of germplasm (landraces, cultivars, elite breeding lines, etc), and provides broader genomic region/allelic coverage with high resolution with-out the need to develop bi-parental mapping populations [6]. Association mapping principally uses linkage disequilibrium (LD) approaches. It is important, however, first to separate LD due to physical linkage from LD due to population structure which can be caused by many natural and artificial factors including the selection and improvement schemes in crop breeding programs [7].

Bayesian analysis using unlinked set of markers has been effectively used to determine population structure by assigning individuals to subpopulations (Q matrix) [8,9]. Clustering and scaling of populations can be used as alternative approaches to determine population structure [10]. To-date, AM has been carried out in many crops and QTLs associated to traits of interest have been identified [11]. For example, in wheat, QTLs associated to kernel size and milling quality [12], grain yield [13], high-molecular-weight glutenins [14], resistance to foliar diseases [13,15,16], Fusarium head blight (FHB) resistance [17], resistance to nodorum blotch [18] and major insect pest resistances [19] have been reported using AM approaches.

In this study, we investigated the association of approximately 3,051 polymorphic diversity array technology (DArT) markers with grain yield, yield-related and quality attributes in 120 elite winter facultative wheat genotypes in order to determine the genetic structure within these wheat genotypes and identify closely associated markers with grain yield and quality for possible use in marker-assisted selection (MAS).

## Material and Methods

### Germplasm and phenotyping

A total of 118 elite facultative winter wheat (FWW) genotypes and two check varieties, Solh and Bezostaya, were used for this study (S1 Table). Genotypes were planted each in a plot size of 6 m^2^ (5 m length, 6 rows at 0.2 m spacing) in two replications using alpha-lattice design in 2011 and 2012 seasons under rain-fed condition at Tel Hadya research field of the International Center for Agricultural Research in the Dry Areas (ICARDA), Syria. The same genotypes were planted each in non-replicated large strip plot of 24m^2^ (20 m length, 6 rows at 0.2 m spacing) at Tel Hadya, Syria for two years (2011 and 2012) under irrigated condition. Trials were managed as per the recommended management practices. Data were recorded for days to heading, day to maturity, plant height (cm), grain yield (kg/ha), 1000 kernel weight, grain color and test weight. All analyses were carried out with GENSTAT [20] software.

### Grain quality analysis

Protein content was assessed using near-infrared transmittance spectrophotometer according to the approved methods of the American Association of Cereal Chemists, AACC, Method No. 39–10 [21]. Dough water absorption (FSA), departure time (FDT), stability time (FST) and mixing tolerance index (MTI) were determined using farinorgraph (Brabender, Germany) according to AACC Method No. 54–21 [21]. White flour samples were used to determine the following rheological properties of dough biaxial extension: tenacity (*P*, maximum overpressure), extensibility (*L*, length of the curve), strength (*W*, deformation energy), and the configuration ratio (*P/L*) with the alveograph (Chopin S.A., Villeneuve la Garenne, France) following the ICC standard method No. 122 [22]. High molecular weight (HMW) glutenin subunits were determined using sodium dodecyl sulfate polyacrylamide gel electrophoresis (SDS-PAGE) [23].

Principal component analysis (PCA) was performed on the correlation matrix, calculated on the data of the quality traits. The PCA analysis was performed using GENSTAT [20] software.

### Genotyping

Genomic DNA was extracted from two weeks old pooled leaf samples collected from five plants per line. The samples were frozen in liquid nitrogen and stored at -80°C before DNA extraction. DNA extraction was carried out according to Ogbonnaya et al. [24], after which 10 μl of a 100 ng μl-1 DNA of each sample was sent to Triticarte Pty. Ltd, Australia (http://www.triticarte.com.au/) as a commercial service provider for whole genome scan using Diversity Arrays Technology (DArT) markers [25]. Three thousand and fifty one DArT markers were used to genotype the 120 wheat genotypes (S2 Table). The markers were integrated into a linkage map by inferring marker order and position from the consensus DArT map [26].

### STRUCTURE analysis

The genetic structure of the 120 genotypes was investigated using 250 unlinked DArT markers distributed across the wheat genome with at least two loci on each wheat chromosome [27]. Bayesian clustering method was applied to identify clusters of genetically similar individuals using the software STRUCTURE version 2.3 [28]. A burn-in length of 10^4^ cycles (to minimize the effect of starting configuration), a simulation run of 10^6^ cycles, and the admixture model option were applied in the Structure program. We chose cluster values (K) ranging from 2 to 24 and six independent runs for each value in order to obtain consistent results. Additionally, the results were further confirmed by the Bayesian Information Criterion for different number of populations obtained using the *adegenet* package [29] for R statistical software [30].

### Linkage disequilibrium

From the complete set of 1743 polymorphic markers, only 1143 markers with known position [26] were selected to perform the linkage disequilibrium analysis using TASSEL V4.3.1 software [31]. Linkage disequilibrium (LD) was estimated as squared allele frequency correlations (*R* ^*2*^), and only *P*-values ≤ 0.01 for each pair of loci were considered significant.

### Association mapping

Grain yield, days to heading, plant height and grain quality data of the 120 elite FWW genotypes and the corresponding DArT data were used for the association mapping. TASSEL version 4.3.1 was used to perform association mapping analysis using both the General Linear Model (GLM) and Mixed Linear Model (MLM) methods. The two different models of GLM: the model with no control for population structure and relatedness (naïve model), and the model with population structure (the Q model) were used. For MLM also two models were used; the model that considers the familial relatedness between accessions (the K model), and the model that takes into account both the population structure and the familial relatedness i.e. the Q+K model [32]. The general equations for GLM and MLM are: *y = Xa + e*; and *y = Xa + Qb+ Zu + e*; respectively; where *y* is vector for phenotypes; *a* is the vector of marker fixed effects, *b* is a vector of fixed effects, *u* is the vector of random effects (the kinship matrix), and *e* is the vector of residuals. *X* denotes the genotypes at the marker; *Q* is the Q-matrix obtained from the STRUCTURE software and *Z* is an identity matrix. Both models were applied with and without considering the fixed effect of the population structure. False discovery rate (FDR) values were calculated at 0.05 according to [33]. The marker is considered significant if its P value is lower than the correspondence FDR value. However, marker alleles with P values ≤ 0.001 in both MLM and MLM-Q models were declared significantly associated with quality parameters and yield and yield related traits since none of the tested markers passed the FDR test.

## Results

### Grain yield and quality performance

There was significant difference in grain yield performance among the genotypes both under rain-fed and irrigated environments. Average yield of the genotypes ranged from 2295 to 4038 kg/ha and 4268 to 7102 kg/ha under rain-fed and irrigated conditions, respectively (Table 1).

**Table 1:** Mean, minimum and maximum values of the different agronomic and quality traits measured on 120 FWW genotypes at Tel Hadya, Syria, 2011–2012.

Trait	Environment	Mean	Max	Min	SED/SD^a^	P-value^b^
Yield (kg/ha)	Rainfed	3157	4038	2295	270	<0.001
	Irrigated	5636	7102	4268	811	0.076
	Average	4396	5381	3499	327	<0.001
Days to heading (days)	Rainfed	145	155	138	2.1	<0.001
	Irrigated	154	165	145	1.92	<0.001
	Average	150	145	155	1.3	<0.001
Days to maturity (days)	Rainfed	180	184	176	1.66	0.005
	Irrigated	196	200	192	2.2	0.013
	Average	188	180	184	1.28	<0.001
Plant height (cm)	Rainfed	73	92.5	60	7.45	0.011
	Irrigated	93.9	115	77.5	7.56	<0.001
	Average	83.5	73	92.5	4.92	<0.001
TKW (g)	Irrigated	32.7	42.4	25.9	3.9	NA
TW (kg/hl)	Irrigated	75.6	81	61.8	2.7	NA
PSI (%)	Irrigated	44.2	65	31	7.4	NA
Protein (%)	Irrigated	13.6	16.1	11.4	1.4	NA
FAB (%)	Irrigated	60.3	68.5	53	3	NA
FDT (min)	Irrigated	3.76	11.5	1	2.1	NA
FST (min)	Irrigated	7.06	22.6	1.9	3.7	NA
MTI (BU)	Irrigated	46.5	95	5	16.2	NA
P (mm H_2_O)	Irrigated	60.1	103	28	17.1	NA
L (mm)	Irrigated	128.3	255	53	33.2	NA
P/L (mm H_2_O/mm)	Irrigated	0.54	1.8	0.1	0.3	NA
W (10^-4^J)	Irrigated	217.6	375	86	57.5	NA

^a^: SED (Standard error of the differences of the means) is indicated for yield, days to heading, days to maturity, plant height; and SD (Standard deviation) is indicated for quality traits: grain protein content, FDT: Farinograph development time, FST: Farinograph stability time, FAB: Farinograph water absorption, MTI: Mixing tolerance index, W: Alveograph strength, P: Alveograph tenacity, L: Alveograph extensibility, P/L: Alveograph configuration ratio, TKW: Thousand kernel weight, TW: Test weight and PSI: Particle size index.

^b^ P-Value: the significance of the differences among the agronomical/quality scores, P > 0.05 means no significant difference among the 120 genotypes for the described trait.

The average yield of the check cultivar (Solh) was 3690 and 5823 kg/ha under rain-fed and irrigated conditions, respectively. The most commonly grown cultivar, Bezostaya, yielded 5200 kg/ha under irrigated conditions. Among the top 20 elite genotypes indicated in Table 2, G19, G30, G56, G70, and G104 out-yielded the check cultivar Solh both under rain-fed and irrigated conditions. G25 and G21 with yield levels of 4038 and 7102 kg/ha are the highest yielding genotypes under rain-fed and irrigated conditions, respectively (Table 2, S1 Table) Significant differences were also observed in days to heading, maturity and plant height under both irrigated and rain-fed conditions. Mean days to heading ranged from 138–155 days under rain-fed conditions and 145–165 days under irrigated conditions. Similarly, mean plant height ranged from 60–93 cm and 77.5–115 cm under rain-fed and irrigated conditions, respectively.

**Table 2:** Mean grain yield and quality performance of the top 20 high yielding genotypes at Tel Hadya, Syria, 2011–2012.

Genotype	Pedigree	Yield under rainfed conditions(kg/ha)	Yield under irrigated conditions(kg/ha)	Yield average(kg/ha)	TKW (g)	TW (kg/hl)	PSI (%)	Protein (%)	FAB (ml)	FDT (min)	FST (min)	MTI (BU)	P (mm H2O)	L (mm)	P/L (mm H2O/mm)	W (10-4J)
G6	PLK/LIRA/5/NAI60/3/14.53/ODIN//[CI13441]/4/GRK79/6/MNCH/7/CROC_1/AE.SQUARROSA (213)//PGO	3068	6567	4817	33.4	76.2	42.9	15.3	65.5	3.2	4.2	50.0	64.0	129	0.5	207
G18	CADET/6/YUMAI13/5/NAI60/3/14.53/ODIN//CI13441/CANON	3337	6621	4979	29.5	77.2	44.0	16.1	64.0	6.2	8.5	33.0	50.0	176	0.3	225
G19	NWT/3/TAST/SPRW//TAW12399.75/6/VEE/TSI//GRK/3/NS55.03/5/C126.15/COFN/3/N10B/P14//P101/4/KRC67	3974	6090	5032	33.1	71.8	43.8	15.6	63.0	6.0	7.5	33.0	56.0	173	0.3	256
G21	PANTHEON/BLUEGIL-2	3660	7102	5381	29.4	71.4	43.8	15.9	68.5	4.0	3.8	47.0	85.0	112	0.8	268
G30	NWT/3/TAST/SPRW//TAW12399.75/6/VEE/TSI//GRK/3/NS55.03/5/C126.15/COFN/3/N10B/P14//P101/4/KRC67	3787	5979	4883	32.8	74.4	46.0	15.0	60.0	11.5	22.6	18.0	63.0	147	0.4	332
G38	SHI#4414/CROWS"//GK SAGVARI/CA8055	3400	6428	4914	35.8	73.8	43.6	14.6	63.5	2.8	2.5	68.0	43.0	121	0.4	126
G39	00247G6-106	3455	6260	4858	34.6	77.0	45.7	15.3	60.5	3.7	7.0	33.0	55.0	119	0.5	209
G40	00247G6-104	3338	6288	4813	34.6	77.2	43.6	15.2	61.5	4.5	5.0	45.0	50.0	120	0.4	185
G41	02106G2-2	3098	6565	4831	29.3	73.8	44.9	15.8	62.0	9.3	22.0	5.0	87.0	108	0.8	373
G42	02429GP1	3489	6554	5022	28.0	73.0	48.8	15.3	61.0	7.7	13.5	26.0	65.0	116	0.6	255
G56	JI5418/MARAS/4/885K4.1//MNG/SDV1/3/1D13.1/MLT	3811	6198	5005	38.1	79.6	59.4	12.6	55.5	1.5	4.8	85.0	38.0	127	0.3	145
G57	1D13.1/MLT//WEAVER/3/RENESANSA	2910	7090	5000	34.0	78.4	36.2	12.8	62.0	2.2	3.2	78.0	41.0	97	0.4	96
G59	4WON-IR-257/5/YMH/HYS//HYS/TUR3055/3/DGA /4/ VPM / MOS	3044	6785	4915	42.4	81.0	56.7	12.5	56.0	1.4	4.1	70.0	33.0	169	0.2	161
G65	PLK/LIRA/5/NAI60/3/14.53/ODIN//[CI13441]/4/GRK79/6/MNCH/7/CROC_1/AE.SQUARROSA (213)//PGO	2948	7065	5006	32.0	78.4	38.8	13.1	59.5	3.4	6.0	44.0	52.0	123	0.4	196
G70	F12.71/SKA//FKG15/3/F483/4/CTK/VEE/5/SHARK/F4105W2.1	3745	5986	4866	40.3	76.2	45.1	12.7	57.5	4.3	10.5	43.0	48.0	128	0.4	221
G84	TOB/ERA//TOB/CNO67/3/PLO/4/VEE#5/5/KAUZ/6/URES/JUN//KAUZ/7/URES/JUN//KAUZ/8/VEE/MJI//2*TUI/3/2*PASTOR	3392	6558	4975	27.9	76.6	59.9	13.7	56.0	5.4	9.0	38.0	45.0	187	0.2	218
G85	BLUEGIL-2/CAMPION	3614	6774	5194	32.0	76.0	46.5	12.8	58.0	5.4	8.0	35.0	66.0	129	0.5	255
G104	TRAP#1/YACO/3/KAUZ*2/TRAP//KAUZ/6/SN64//SKE/2*ANE/3/SX/4/BEZ/5/JUN/7/SHARK-1	3733	5964	4848	30.1	74.6	39.9	13.0	59.5	4.9	9.3	28.0	68.0	142	0.5	273
G105	CAR422/ANA//YACO/3/KAUZ*2/TRAP//KAUZ/4/BUCUR/5/BUCUR	3617	6063	4840	29.9	75.8	39.9	13.0	60.5	4.0	9.2	25.0	73.0	127	0.6	257
G107	KARL/NIOBRARA//TAM200/KAUZ/3/TAM200/KAUZ	3520	7051	5286	27.5	77.2	59.4	12.3	55.0	1.2	5.8	61.0	48.0	130	0.4	166
SOLH	OK-82282//BOBWHITE/NEELKANT	3690	5823	4757	34.8	78.4	38.6	13.3	58.5	6.0	12.0	32.0	61.0	160.0	0.4	291.0
BEZOSTAYA	LUTESCENS-15/SKOROSPELKA-2	-	5200	-	32.1	78.0	40.0	16.6	66.0	6.0	12.4	40.0	116.0	117.0	1.0	476.0

Grain quality traits were evaluated under irrigated condition. Mean protein content ranged from 11.4 to 16.1%. Farinograph development time (FDT), Farinograph stability time (FST) and Alveograph strength (W) ranged from 1 to 11.5 min, 1.9–22.6 min and 86–375 Jx10^-4^, respectively (Table 1).

The analysis of the rheological behavior of the dough through the alveograph showed that the check Bezostaya performed better in dough strength, dough tenacity and protein content as compared to the 118 FWW genotypes. The top 20 high yielding genotypes showed a very close performance in quality traits to the best checks. Additionally, the best 20 lines showed significantly higher (*p*<0.05) average grain protein content, Farinograph development time and stability time than the other 98 FWW genotypes (Table 2).

**Fig 1:**
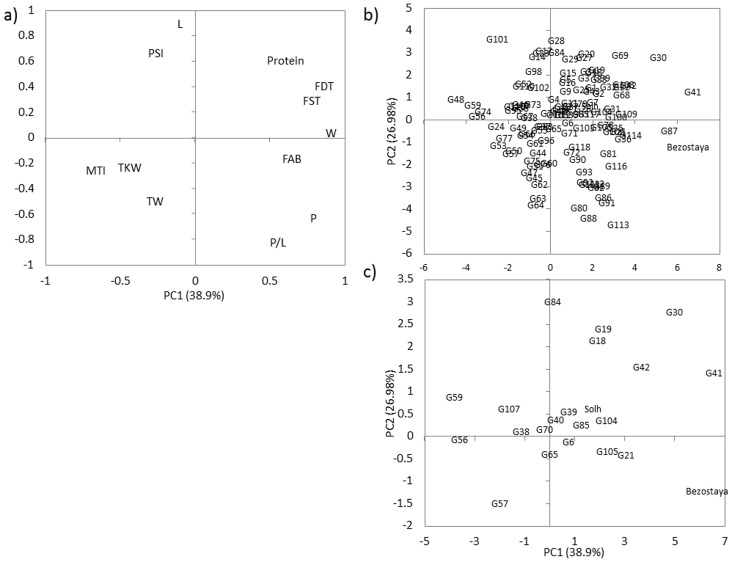
Biplot of principal component analysis. (A) Eigenvalues of the correlation matrix symbolized as vectors representing the quality traits. (B) The 118 Facultative and Winter Wheat ICARDA lines and Bezostaya and Solh are plotted on the plane determined by the first two PC. (C) The best 20 lines are plotted on the plane determined by the first two PC. Protein: Grain protein content, FDT: Farinograph development time, FST: Farinograph stability time, FAB: Farinograph water absorption, MTI: mixing tolerance, W: Alveograph strength, P: Alveograph tenacity, L: Alveograph extensibility, P/L: Alveograph configuration ratio, TKW: Thousand kernel weight, TW: Test weight.

Principal Component Analysis (PCA) of the 12 quality parameters in 120 FWW genotypes indicated that protein content, FDT, FST and W are very important traits in discriminating the genotypes (Fig 1A). Based on the results obtained, a wide range of quality traits combinations was found among the set of 120 genotypes (Fig 1B). Bezostaya, G87, G41, G30, and G42 are situated in the positive sense of the protein content, FDT, FST, FAB and W vectors indicating that they performed particularly well at these quality traits (Fig 1B and 1C).

**Fig 2:**
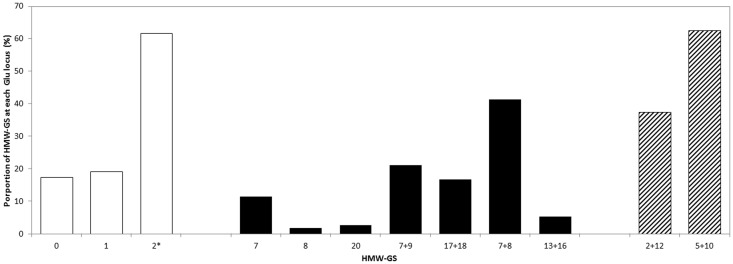
Proportion of the elite facultative and winter wheat lines carrying a specific allele at each *Glu* loci. *Glu-A1* open bars, *Glu-B1* closed bars and *Glu-D1* striped bars.

High molecular weight glutenin subunit (HMW-GS) composition analysis showed that more than 80% of the genotypes carry Glu-A1 or Glu-A2* alleles. Regarding the HMW-GS encoded in Glu-B1, 7+8, 7+9 and 17+18 were the most common subunits accounting for 41.2, 21.1 and 16.7%, respectively (Fig 2). More than 60% of the 120 genotypes possess the 5+10 allele at Glu-D1 locus. Most of the 20 high yielding genotypes possess the 2* Glu-A1, 7+8 Glu-B1 and 5+10 Glu-D1 alleles.

### DArT markers statistics

All genotypes were tested with 3,051 DArT markers. A total of 1734 DArT markers were selected for analysis due to their polymorphism. Five hundred and seventy nine markers were distributed on the A genome, while a total of 708 and 316 polymorphic markers were distributed on the B and D genomes, respectively. The position of 131 polymorphic markers was unknown. The average P value, call rate and polymorphism information content (PIC) for all the markers was 78.5, 0.3 and 90.3, respectively.

### Population structure and linkage disequilibrium

**Fig 3:**
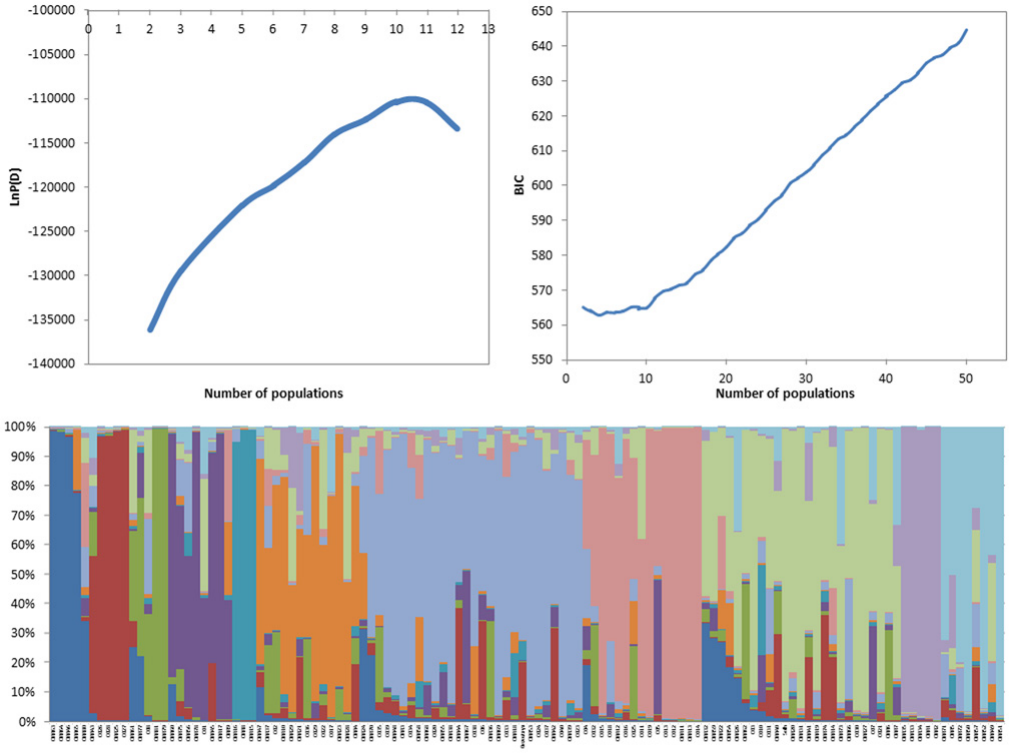
Population structure among genotypes. A) Plot of the average logarithm of the probability of data likelihood [Ln P(D)], as a function of the number of assumed subgroups (k), with K allowed to range from 2 to 12. B) Plot of the Bayesian Information Criterion for each population number from 1 to 50 C) The proportion of the genome of each individual originating from each inferred population (a total of 11 and each color represent a single population)

As indicated in Fig 3, the K values (number of populations) steadily kept on increasing until K = 11 indicating that the 120 FWW genotypes in this study are clustered into 11 subpopulations. Genetic variation among the 11 identified sub-populations was tested using F-statistics, estimated from pairwise comparisons as a measure of genetic distance between subpopulations. F-statistics values between sub-populations were significant (P = 0.01) and ranged from 0.05 to 0.97, supporting the existence of genetic structure. Clusters 6, 7, and 8 consisted of 28, 15, and 24 genotypes, respectively. The other clusters consisted of less than 8 genotypes. The check cultivars Bezostaya and Solh were placed in subpopulations 7 and 9, respectively. The top 20 high yielding genotypes were distributed across the seven subpopulations except in subpopulations 1, 3, 5 and 10.

Linkage disequilibrium was calculated separately for locus pairs within the same chromosomes and between chromosomes. There were 41192 (6.9%) inter-chromosomal pairs of loci showing significant LD (p < 0.01), 1715 (4.2%) of which had *R* ^*2*^ > 0.2. Of the intra-chromosomal locus pairs, 9917 (24.8%) had a significant LD of which 5044 (50.9%) had R^2^ > 0.2.

**Fig 4:**
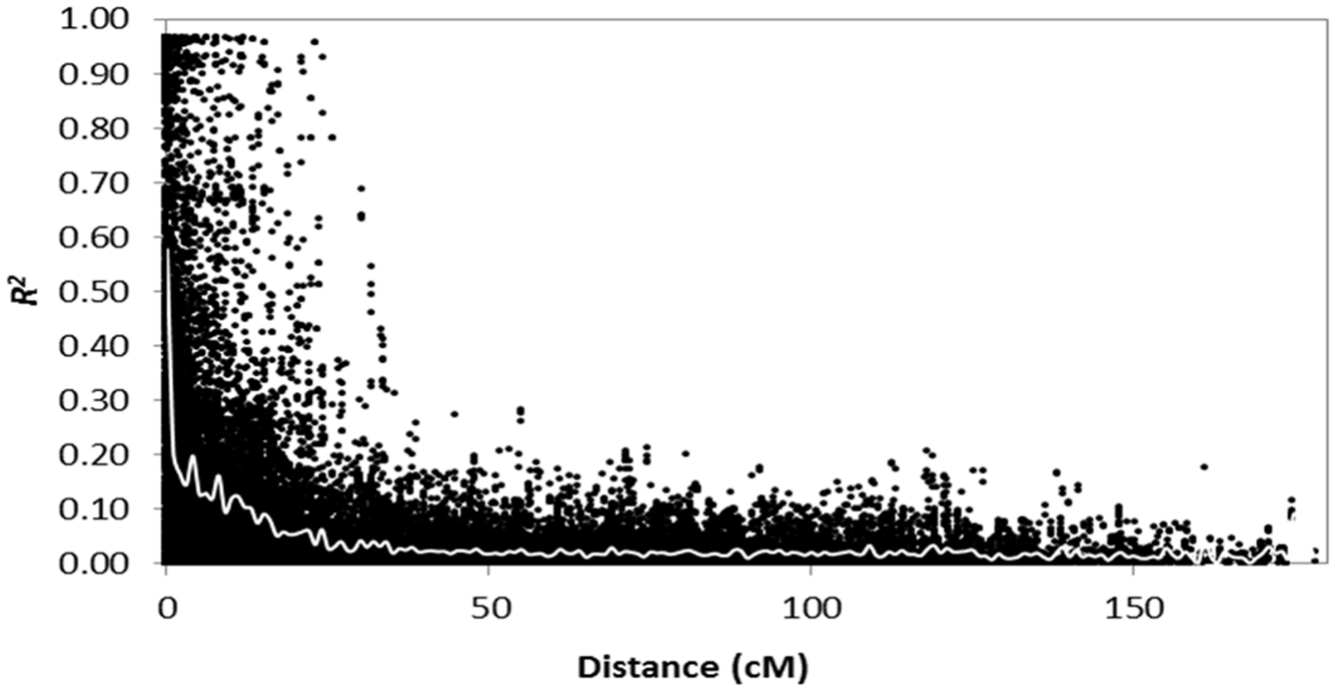
Decline of LD as measured by R^2^ against genetic distance

Intra-chromosomal locus pairs have a higher mean R^2^ value (0.10) than inter-chromosomal locus pairs (0.02). The scatter plots of LD (*R* ^*2*^) as a function of the inter-marker distance (cM) within the same chromosome for all genotypes indicated a clear LD decay with genetic distance (Fig 4). LDs with *R* ^*2*^ > 0.2 extended to distances up to 35 cM suggesting that the mapping resolution using these genotypes would generally be well below 35 cM. Genome wide *R* ^*2*^ estimates declined rapidly from 0.58 for markers with 0 interval distance to 0.13 within 5 cM of genetic distance across all chromosomes.

### QTLs associated with yield and yield related traits

**Fig 5:**
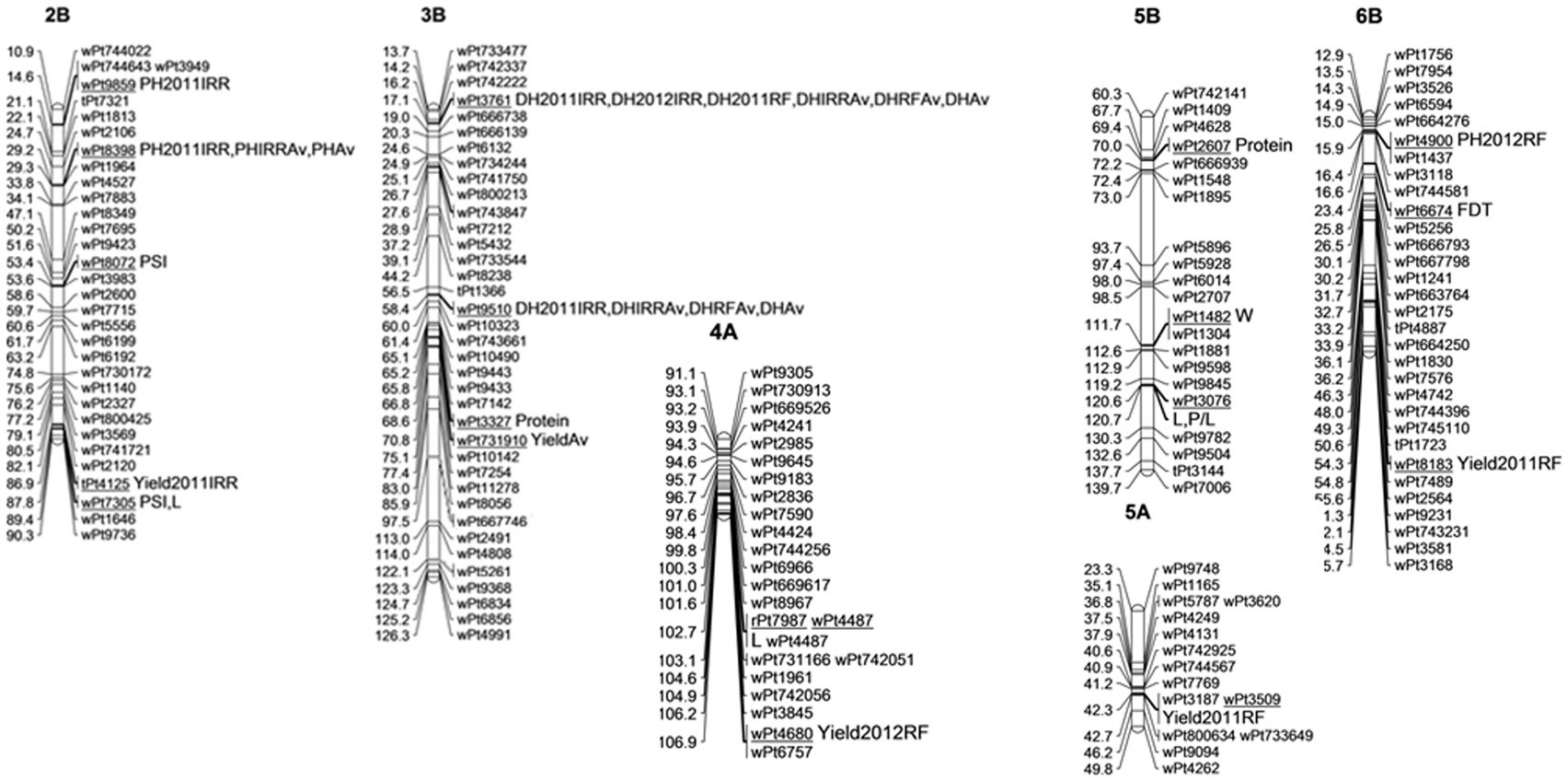
Association mapping profiles of wheat agronomic performance and quality attributes. Consensus linkage maps are based on information from Huang et al. (2012). On the left, values are genetic distance in centimorgans (cM). On the right, diversity array technology markers (underlined) are significantly associated with resistance to wheat quality attributes. On the far right side, the agronomic performance and quality attributes associated are indicated. Protein: Grain protein content, FDT: Farinograph development time, FST: Farinograph stability time, L: Alveograph extensibility, P/L: Alveograph configuration ratio, W: Alveograph strength. DH2011IRR: Days to heading recorded in 2011 under irrigated conditions; DH2011RF: Days to heading in 2011 under rainfed conditions; DH2012IRR: Days to heading in 2012 under irrigated conditions, DHIRRAv: Average days to heading in the irrigated trials, DHRFAv: Average days to heading in the rainfed trials, DHAv: Average days to heading accross environments; PLH2011IRR: Plant height in 2011 under irrigated conditions; PH2012RF:Plant height in 2012 under rainfed conditions, PHIRRAv: Average plant height in the irrigated trials, PLHAv: Average plant height accross environments; YLD2011IRR: Yield in 2011 under irrigated conditions; YLD2011RF: Yield in 2011 under rainfed conditions, YLD2012RF: Yield in 2012 under rainfed conditions. YieldAv: Average yield accross environments.

DArT markers significantly associated with grain yield and yield related traits under rain-fed and irrigated conditions were identified (Table 3, Fig 5).

**Table 3:** Chromosome location, MAF, P, FDR, and R^2^ values of significantly associated DArT markers with grain yield and other agronomic traits under rain-fed, irrigated, average (rain-fed &irrigated) conditions in 2011 and 2012 season at Tel Hadya, Syria. For each marker-trait association, references of published QTL in the same chromosome are also included.

Trait	Environment/Season	Marker	Chromosome	Position	MAF	*P*	*FDR*	*R* ^*2*^	*References*
Yield	Irrigated/2011	tPt4125	2B	86.9	54.39	6.60E-04	2.88E-05	0.13	[13]
	Rainfed/2011	wPt0298	2D	NA	58.93	1.38E-04	2.88E-05	0.17	[13, 34]
		wPt3509	5A	42.3	68.18	5.54E-04	8.65E-05	0.14	[13, 35, 36]
		wPt8183	6B	54.3	78.45	5.04E-04	5.77E-05	0.14	[13, 35]
	Rainfed/2012	wPt4680	4A	106.9	81.58	7.12E-04	2.88E-05	0.12	[13, 37, 35]
	Rainfed/Average (2011–2012)	wPt731910	3B	70.8	75.63	9.71E-04	2.88E-05	0.09	[13, 34]
Days to heading	Irrigated/2011	wPt3761	3B	17.1	61.61	3.11E-04	5.77E-05	0.15	[38]
		wPt9510	3B	58.4	58.77	2.87E-04	2.88E-05	0.15	[38]
		wPt2507	5B	NA	87.29	9.03E-04	8.65E-05	0.13	[39]
	Irrigated/2012	wPt2938	3A	53	60.34	9.96E-04	5.77E-05	0.12	[35, 38]
		wPt3761	3B	17.1	61.61	2.92E-04	2.88E-05	0.15	[38]
	Rainfed/2011	wPt2938	3A	53	60.34	4.95E-04	5.77E-05	0.14	[38]
		wPt3761	3B	17.1	61.61	1.19E-04	2.88E-05	0.17	[38]
	Irrigated/Average (2011–2012)	wPt3761	3B	17.1	61.61	1.76E-04	2.88E-05	0.16	[38]
		wPt9510	3B	58.4	58.77	3.51E-04	5.77E-05	0.15	[38]
		wPt2507	5B	NA	87.29	8.11E-04	8.65E-05	0.13	[39]
	Rainfed/Average (2011–2012)	wPt6422	3A	177.6	77.27	9.36E-04	8.65E-05	0.13	[38]
		wPt3761	3B	17.1	61.61	5.00E-04	2.88E-05	0.14	[38]
		wPt9510	3B	58.4	58.77	7.88E-04	5.77E-05	0.13	[38]
	Average (Irrigated-Rainfed/2011-2012)	wPt3761	3B	17.1	61.61	1.01E-04	2.88E-05	0.17	[38]
		wPt9510	3B	58.4	58.77	3.66E-04	5.77E-05	0.15	[38]
Plant height	Irrigated/2011	wPt9859	2B	14.6	77.59	2.27E-04	5.77E-05	0.16	[35]
		wPt8398	2B	29.2	53.7	2.20E-04	2.88E-05	0.16	[35]
	Rainfed/2012	wPt4900	6B	15.9	57.39	2.26E-04	2.88E-05	0.15	
	Irrigated/Average (2011–2012)	wPt8398	2B	29.2	53.7	3.37E-04	2.88E-05	0.15	[35]
		wPt9067	4B	68.1	85.47	9.64E-04	5.77E-05	0.13	[35, 36]
	Average (Irrigated-Rainfed/2011-2012)	wPt8398	2B	29.2	53.7	9.10E-04	2.88E-05	0.13	[35]
		wPt1601	7A	160.3	73.28	9.99E-04	5.77E-05	0.13	[40]

MAF: Major allele frequency; FDR: False discovery rate

The DArT markers *wPt0298* (2D), *wPt3509* (5A) and *wPt8183* (6B) were significantly associated with yield under rain-fed conditions during the 2011 season. During the 2012 season in rain-fed condition, only *wPt4680* (4A) was significantly associated with yield explaining about 12% of the variation. Based on the two year’s mean grain yield data under rain-fed conditions, *wPt731910* (3B) was found to be associated with grain yield with R^2^ value of 9%. Under irrigated condition during the 2011 season, only *tPt4125* on chromosome 2B was significantly associated with yield explaining about 13% of the variation. A total of 15 significantly marker-trait associations were found with days to heading in rain-fed and irrigated conditions during 2011 and 2012 seasons and corresponding averages. During the 2011 season under rain-fed condition, *wPt3761* on chromosome 3B and *wPt2938* on chromosome 3A were significantly correlated with days to heading covering 17 and 14% of the variation, respectively. Similarly, markers significantly associated with plant height both under rain-fed and irrigated conditions were identified (Table 3).

### QTLs associated with grain quality traits

The implementation of the MLM using the Q+K model showed that out of the 1734 DArT markers, only 20 markers showed significant association (*p*≤0.001) with quality traits (Table 4, Fig 5).

**Table 4:** Chromosome location, MAF, P, FDR and R^2^ values of significantly associated DArT markers with grain quality traits at Tel Hadya, Syria, 2011–2012. For each marker-trait association, references of published QTL in the same chromosome are also included.

Trait	Marker	Chromosome	Position	MAF	*P*	FDR	R^2^	References
TKW	wPt6122	1A	23.8	52.6	7.20E-04	5.77E-05	0.11	[41, 42]
	wPt2847	1A	117.2	59.8	3.09E-04	2.88E-05	0.13	[41, 42]
	wPt5892	7B	192.4	77.1	8.51E-04	8.65E-05	0.11	[42]
TW	wPt667984	1A	64.9	73.7	5.86E-04	8.65E-05	0.14	[42]
	wPt4801	1A	87.9	73.5	1.89E-04	5.77E-05	0.16	[42]
	wPt3524	6A	20.2	68.6	3.19E-05	2.88E-05	0.2	
Protein	wPt3327	3B	68.6	75.3	1.51E-04	5.77E-05	0.15	[43, 44]
	wPt2607	5B	70	67.4	9.86E-05	2.88E-05	0.16	[43]
PSI	wPt8072	2B	53.4	72.4	9.50E-04	5.77E-05	0.13	
	wPt7305	2B	87.8	87.9	5.99E-05	2.88E-05	0.18	
FDT	wPt667413	5D	NA	75.9	7.46E-04	5.77E-05	0.13	
	wPt6674	6B	23.4	82.1	4.46E-04	2.88E-05	0.14	
FST	wPt733835	1D	86.5	54.8	2.84E-04	1.15E-04	0.14	[44, 45, 46]
	wPt665749	1D	NA	69.5	3.74E-04	1.73E-04	0.14	[44, 45, 46]
	wPt744943	2A	18.7	88.1	3.09E-04	1.44E-04	0.14	
	wPt668044	2D	86.5	68.4	5.90E-04	3.75E-04	0.13	
	wPt743847	3B	27.6	58.1	4.08E-04	2.02E-04	0.14	[45]
	wPt667746	3B	97.5	81.2	4.81E-04	2.88E-04	0.13	[45]
	wPt5261	3B	122.1	80.5	1.85E-04	2.88E-05	0.15	[45]
	wPt740798	3D	NA	88.5	5.82E-04	3.46E-04	0.13	
	wPt734157	6A	23.3	78	2.59E-04	8.65E-05	0.15	
	wPt741290	6A	23.3	77.6	6.38E-04	4.04E-04	0.13	
	wPt0228	6A	NA	83.9	1.97E-04	5.77E-05	0.15	
	wPt732125	6A	NA	79.3	5.05E-04	3.17E-04	0.13	
	wPt2883	7B	NA	82.2	4.27E-04	2.60E-04	0.14	
	wPt744866	7D	1.6	83.9	4.09E-04	2.31E-04	0.14	
	wPt663992	7D	1.9	81.7	9.77E-04	4.33E-04	0.12	
MTI	wPt733835	1D	86.5	54.8	4.63E-05	2.88E-05	0.18	[44,46]
P	wPt742908	7D	NA	85.5	5.17E-04	2.88E-05	0.14	[47]
P/L	wPt3076	5B	120.6	68.5	6.70E-04	8.65E-05	0.13	
	wPt742908	7D	NA	85.5	2.18E-04	2.88E-05	0.15	
	wPt744477	7D	1.5	89.7	2.90E-04	5.77E-05	0.14	
L	wPt7305	2B	87.8	87.9	3.10E-04	5.77E-05	0.13	[45, 47]
	rPt7987	4A|7A	102.7, 10.0	50.9	1.37E-04	2.88E-05	0.14	[45]
	wPt4487	4A|7A	102.7, 10.0	54.8	4.53E-04	1.15E-04	0.12	[45]
	wPt3076	5B	120.6	68.5	3.78E-04	8.65E-05	0.13	[47]
W	wPt3177	1B	NA	82	5.17E-04	5.77E-05	0.14	[45, 47, 48]
	wPt733835	1D	86.5	54.8	4.81E-04	2.88E-05	0.14	
	wPt1482	5B	111.7	68.7	4.97E-04	8.65E-05	0.14	[47]

FDR: False discovery rate, MAF: Major allele frequency, Protein: Grain protein content, FDT: Farinograph development time, FST: Farinograph stability time, FAB: Farinograph water absorption, MTI: Mixing tolerance index, W: Alveograph strength, P: Alveograph tenacity, L: Alveograph extensibility, P/L: Alveograph configuration ratio, TKW: Thousand kernel weight, TW: Test weight PSI: Particle size index.

Seven of these markers were located on the A genome while 9 and 4 markers were located on the B and D genomes, respectively. Four of the 20 markers identified showed association with two quality traits resulting in 24 marker-quality trait associations. Out of the total number of markers significantly associated with grain quality, *rPt7987* and *wPt4487* were present on both 4A and 7A chromosomes; two DArT markers do not have known positions, while 16 DArT markers with known position were associated with ten quality parameters on 9 different chromosomes (Table 4). A total of four QTL for dough extensibility (L); three QTLs for each of dough strength (W), configuration ratio (P/L), thousand kernel weight (TKW) and test weight (TW), twoQTLs each for farinograph departure time (FDT), particle size index (PSI) and protein content and one QTL each for mixing tolerance index (MTI) and tenacity (P) were identified (Table 4). Markers *wPt2607* (5B) and *wPt3327* (3B) were significantly associated with protein content covering 16 and 15% of the variations, respectively. Three markers: *wPt733835* (1D), *wPt1482* (5B) and *wPt3177* (1B) were associated significantly with alveograph strength (W) with R^2^ values of 14% each. Marker *wPt742908* with unknown position showed significant association with P/L and P while marker *wPt3076* on 5B showed association with P/L and L (Table 4).

## Discussion

### Combining yield potential with drought tolerance

As water is becoming scarce even in the irrigated areas, ICARDA’s germplasm development approach aims to combine high yield potential with drought tolerance so that wheat genotypes targeted for irrigated areas can cope with temporary drought periods. Similarly, this approach enables the wheat breeding program to minimize and maximize yield gains during drought and good seasons, respectively, for the rain- fed production system. To this end, the wheat breeding program at ICARDA in collaboration with the International Winter Wheat Improvement Program (IWWIP) have made rigorous efforts and identified elite facultative/winter wheat genotypes which combines high yield potential and drought tolerance with acceptable grain quality and resistance to yellow rust [15]. The outstanding performance of these genotypes both under rain-fed and irrigated environments indicates the wide adaptation of the genotypes. Wide adaptation of wheat genotypes have been reported by many authors [2,49–52]. In this breeding approach, focus is on empirical selection of genotypes that grow faster and establish complete canopy in winter under lower evaporative demand and complete the cropping cycle as early as possible [2].

The quantity and quality of the gluten proteins are major factors determining wheat end-use quality. Gluten proteins are the main components of the gluten matrix and play the main role defining its properties [53]. Among the gluten proteins, high molecular weight glutenin subunits (HMW-GS) are of particular interest in bread wheat due to their large influence over the rheological properties of dough [23,54,55]. The present study showed that the elite genotypes tend to carry HMW-GS that promote dough strength and improve end-use quality. It has been established that low proportion of the null alleles encoded in Glu-A1 have a negative effect on *W* [56,57]. Similarly high proportion of 7+8, 17+18, 13+16 alleles on *Glu-B1* [55,56] and the 5+10 HMW-GS *on Glu-D1* [58–60] have positive effect on dough quality. Unlike most of the elite genotypes, the check cultivars, Solh and Bezostaya, possess HMW-GS that promote dough strength and end-use quality. Bezostaya´s exceptionally high protein content, the second main factor controlling the gluten matrix properties, has probably contributed to its high quality values. The grain quality result in the present study is in line with the negative relationship between grain yield and grain protein content reported in previous studies [34,61,62]. Increases in grain yield are usually accompanied by decreases in grain protein as a result of a dilution of nitrogen compounds when carbohydrate deposition increases during photosynthesis [34].

### Marker-Trait Associations

In the present study, several markers associated with grain and dough quality attributes were identified. Sixteen markers were found to be associated with yield or yield related traits. From these, six markers on chromosomes 2D, 2B, 3B, 4A, 5A, and 6B were directly related to grain yield. Consistent QTL’s associated to yield on chromosome 2D have previously been reported [13,37]. The photoperiod response-related gene *Ppd-D1* and the reduced height gene *Rht8* were previously mapped on chromosome 2D and have a main effect on yield and plant adaptation [63]. Li et al. [35] also reported a QTL at 6B associated with grain yield. Major QTLs associated with grain yield on chromosome 4A were identified in cultivar Dharwar-Dry [64]. Lopes et al [43,45] also identified major QTLs on 6A in Krichauff from the Berkut/Krichauff population, and on 7A from RAC875/Excalibur as well as other QTLs on 1B, 3B, 4A and 4B from Seri/Babax population. In our study, five markers associated with plant height were identified in chromosome 2B, 4B, 6B and 7A. The markers in 2B are probably linked to the same QTL since they are at close distance from each other. The five QTL’s associated with plant phenology in this study were located on chromosomes 3A, 3B and 5B. The group 3 chromosomes have been reported to be associated with vernalization (3B) and earliness *per se* (3A) [38]. The QTL found in chromosome 5B is probably linked to the *Vrn-B1* gene reported in the same chromosome.

Our results indicate the presence of both common and environment-specific QTL in our elite winter wheat germplasm for yield, days to heading and height. Those QTL will facilitate for better planning for future crosses depending on the purpose of the breeding program and the targeted environments. Moreover, the validation and pyramiding of QTL detected under a range of environmental conditions such as the ones mapped in chromosome 2B, 3A, 3B and 5B will be major aims in our future research.

Out of the 24 marker-quality trait associations identified, two involved grain protein content. These markers, *wPt3327* and *wPt2607*, on chromosomes 3BL and 5BL, showed no significant association (*p*>0.05) between them and TKW or yield performance at any environment, suggesting that these QTL’s may not have a negative impact on these traits. Heo and Sherman [65] also identified two QTL’s on 3BL and 5BL related to grain protein content in a Choteau by S-Yellowstone recombinant inbred line (RIL) population involving a cross between a winter wheat, donor of both favourable alleles and different from the varieties involved in the present study, and a spring wheat variety.

Fourteen marker-trait associations were identified regarding mixing and rheological properties. Some of them involved the same marker associated with different traits, probably due to the high correlation between some of the mixing and rheological properties [66]. Six markers were located on the three homeologous chromosome 1, i.e. 1A, 1B and 1D. Two of these 6 markers (*wPt3177* and *wPt733835*) were associated with traits related to dough rheological and mixing properties like dough strength and MTI while the four markers on chromosome 1A showed association with test weight and thousand kernel weight. The large effect of the *Glu-1*, *Glu-3* and *Gli-1* loci complexes over dough properties is widely known [58,60] and their physical position in chromosomes 1A, 1B and 1D [41,42,46,66] suggest that they may be behind some of these associations. Also, some QTL have been described in the homeologous group 1 chromosomes associated to rheological [66] and mixing properties [66,67]. The four markers identified on chromosome 1A associated with test weight and thousand kernel weight are probably linked to different QTL since independent QTL for seed morphology in chromosome 1A have already been reported [44,68]. In the present study, markers associated with rheological and mixing properties were found on chromosomes 2B, 4A/7A, 5B, 5D, 6B and 7D. This is in line with previous reports [66,67,69,70]. Further research using bi-parental populations is recommended in order to validate and determine the similarity of the QTL identified from the present and previous studies. The elite genotypes with high yield potential, drought tolerance and acceptable grain quality traits are recommended for potential direct release and/or use as parents after local adaptation trials by the NARSs of respective countries.

## Supporting Information

S1 Table Summary of the yield performance under rainfed and irrigated conditions and quality parameters for the 118 elite facultative and winter wheat varieties. Yield under rainfed and irrigated conditions are both means across 2 field experiments carried out in Tel Hadya in 2011 and 2012.
(XLSX)

S2 Table Allelic pattern for the 118 elite lines and Bezostaya and Solh for the 67 marker-trait associations found and the rest from the 3051 markers used in the study. Yield average across 4 environments (2011-2012/irrigated and rainfed in Tel Hadya, Syria) and mean yield under rainfed and irrigated conditions are also shown.
(XLSX)
